# Angiotensin receptor blockers (ARBs) in hypertension patients: earlier use of these bettertolerated medications is warranted

**Published:** 2010-04

**Authors:** J Aalbers

**Affiliations:** Special Assignmetns Editor

## Introduction

All patients with hypertension are at increased risk for vascular events and hypertension is widely regarded as one of the most important risk factors for cardiovascular disease.^1^ At all ages, there is a positive and graded relationship between usual blood pressure and the risk of cardiovascular and stroke mortality.^2^

The role of the renin–angiotensin– aldosterone system (RAAS), even in the early stages of the cardiovascular continuum, is well-established, with positive results being obtained in clinical trials of cardiovascular event reduction using specific RAAS blockers.

In hypertension management, tolerability is key to patient compliance and the long-term reduction of cardiovascular events. ARBs with placebo-like tolerability and efficacy in reducing cardiovascular and cerebrovascular events should be favoured in guidelines.^3^

ARBs reduce blood pressure at least to the same extent as ACE inhibitors and more so in some cases. For example, the largest study undertaken to compare an ARB with an ACE inhibitor, the 1 600-patient Prospective Randomized Investigation of the Safety and efficacy of Micardis versus ramipril using Ambulatory blood pressure monitoring (PRISMA) study found greater blood pressure reductions from telmisartan than ramipril.^4^

There is significant evidence of within-class differences among ARBs with respect to their plasma half-lives, lipophilicity and receptor-binding affinity. Drugs in the ARB class also differ in their antihypertensive efficacy.^5^ Clinicians should evaluate these aspects carefully when selecting ARBs for individual patients.

There is also increasing evidence that, as with ACE inhibitors, ARBs confer benefits beyond their blood pressurelowering effects by reducing morbidity and mortality in cardiovascular, renal and cerebrovascular outcomes through renin–angiotensin system (RAS) blockade.^6^

## Hypertensive patients at increased risk

Telmisartan is the only ARB that has demonstrated therapeutic equivalence to the ACE inhibitor ramipril in hypertensive patients at increased vascular risk. The patient population in this study (ONTARGET) is of particular interest as it is representative of the majority of hypertensive patients seen in everyday clinical practice.

The findings from this study showed that telmisartan 80 mg per day was as efficacious as the proven dosage of ramipril (10 mg/day) in reducing risk of cardiovascular death, myocardial infarction, stroke and hospitalisation for heart failure in a broad cross section of highrisk cardiovascular patients. It achieved these results with far fewer side effects, resulting in significantly fewer patients discontinuing therapy.^7^

## Implications for practice

In clinical practice, ARBs are linked to greater patient adherence and to better blood pressure control. For this reason, first-line ARBs can offer improved efficacy, greater compliance and reduced healthcare utilisation, which offset higher acquisition costs.

Importantly, all ACE-intolerant patients who are at risk of diabetes, myocardiaI infarction, stroke or peripheral arterial disease should be switched to the ARB with proven benefit.
